# Models of fertilization kinetics

**DOI:** 10.1098/rsos.150175

**Published:** 2015-09-16

**Authors:** Jussi Lehtonen

**Affiliations:** Evolutionary Biology, Zoological Institute, University of Basel, Vesalgasse 1, CH-4051 Basel, Switzerland

**Keywords:** fertilization kinetics, gamete, isogamy, anisogamy, sperm, egg

## Abstract

Fertilization functions describe how the number of realized fertilizations depends on gamete numbers or density. They provide insight into the fertilization process, and are important components in models on the evolution of reproductive and sex-specific traits. Existing fertilization functions generally examine the proportion of fertilized eggs as a function of sperm numbers or density in a given fertilization environment. Because these functions have been developed for species with highly diverged gametes, there is an inbuilt (and well justified) asymmetry in them: they treat eggs and sperm, and therefore the two sexes, differently. Although very useful, such functions cannot therefore be used to consistently model early stages in the evolution of the two sexes, or extant species where sex-specific gamete sizes and numbers are similar. Here, I derive fertilization functions that describe the fertilization process without making prior assumptions about the two sexes, and are therefore consistent under any level of gamete dimorphism. These functions are compatible with simpler fertilization functions under appropriate conditions. Such functions can be particularly useful in understanding the early stages in the differentiation of the two sexes, as well as its consequences, where the gametes from the two sexes should be treated on an equal basis.

## 1. Introduction

The process of fertilization is of fundamental importance in the evolution of all sexually reproducing species because an individual's fitness is inexorably linked to the successful fertilization of its gametes. Aspects of the fertilization process can have far-reaching evolutionary implications via sperm limitation [1,2], sperm competition [3,4], as well as an interplay between these two factors [5–9].

Many properties of the fertilization process, particularly sperm limitation, can be mathematically captured in fertilization functions (while sperm competition is usually a separate model component). These are mathematical functions that describe how the number or proportion of fertilized gametes relates to gamete numbers or density. The simplest fertilization function, and one that is often (either explicitly or implicitly) used in evolutionary models, is one that assumes that all gametes of the less numerous type (e.g. eggs) are fertilized. More advanced, explicit fertilization functions model the relationship between sperm numbers/density and the proportion of fertilized eggs; often these are negative exponential functions [10,11] or combinations thereof [12], but other saturating functions have also been used (e.g. [7,8]).

However, these existing functions generally treat female and male gametes differently; in other words, they assume strongly diverged male and female gametes. In particular, sperm concentration is assumed to be orders of magnitude bigger than egg concentration (e.g. [10,11]). Additionally, multiple gamete collisions are usually modelled in a way that assumes that eggs are much larger than sperm [10,12]. These are perfectly reasonable assumptions for the intended purposes of these models: modelling the fertilization process in species with strongly diverged sexes, where male gametes can be a tiny fraction of the size of female gametes. However, they are unrealistic assumptions when modelling the early stages of the differentiation of the sexes, or any species where the number of male and female gametes can be very similar.

One of the clearest examples where existing fertilization functions are not sufficient is the evolution of anisogamy (i.e. gamete dimorphism), because models of this transition must be able to consistently handle scenarios where gamete dimorphism can vary from very low to high values. Gamete competition and gamete limitation are two evolutionary pressures that have been argued to drive the evolution of anisogamy; gamete size divergence via gamete limitation was first modelled in the 1930s [13], and via gamete competition (which has become widely accepted as an explanation for anisogamy) in the 1970s [14] (for reviews, see [15–17]). A later model argued that in the context of anisogamy evolution, these two evolutionary pressures are not mutually exclusive, but rather two aspects of one evolutionary process [18]. However, this required a relatively complex model structure. Self-contained fertilization functions that are consistent with low anisogamy ratios and that allow for variable levels of gamete limitation would significantly simplify the modelling of evolutionary processes where both gamete competition and gamete limitation (or sperm competition and sperm limitation) may be important.

Here, I derive fertilization functions that must fulfil the following criteria. (i) They must be unbiased and neutral in the sense that no prior assumptions are made about the relative abundance of male and female gametes. Instead, any consequences of a numerical imbalance must arise from the model. (ii) They must be consistent in that the total number of fertilizations by male gametes must be exactly the same as the total number of fertilizations by female gametes, regardless of any initial asymmetry in total gamete numbers. This symmetry in the reproductive fitness of the sexes is commonly called the Fisher condition [19]. Although seemingly obvious, it has many surprisingly complex and far-reaching evolutionary consequences (e.g. [19,20]), and failing to take it into account can lead to misleading and erroneous results. (iii) Finally, the new functions must converge with previously derived fertilization functions under appropriate conditions.

## 2. Models

The fertilization functions I seek must account for depletion of both gamete types due to fertilizations, as well as gamete mortality (the latter must here be interpreted in a general sense: it covers all the ways in which gametes are removed from the pool of available gametes, except gamete unions). The general framework that I use for deriving the functions is an initial value problem, which is composed of a pair of coupled differential equations that describe the fertilization process, and the initial number of gametes at the time of gamete release. *x*_*t*_ and *y*_*t*_ refer to the number of gametes of type *x* and type *y* (which could be male and female, or vice-versa) at any time *t* after release. To avoid excessive subscripts in the equations, I will use the notation *x*_0_=*x* and *y*_0_=*y* to denote the initial numbers of gametes; see table 1 for notation. In general form, the equations are as follows:

2.1 $$\left\{ \begin{array}{l} \frac{dx_{t}}{dt}=-ax_{t}y_{t}-\mu_{x}x_{t}\\ \frac{dy_{t}}{dt}=-ax_{t}y_{t}-\mu_{y}y_{t} \end{array}\right.\;\text{and}\;\left\{ \begin{array}{l} x_{0}=x\\ y_{0}=y \end{array}\right..$$

These equations roughly translate to:

starting from initial numbers *x*_0_=*x* and *y*_0_=*y*, the rates at which gametes of type *x* and *y* are depleted (*dx*_*t*_/*dt* and *dy*_*t*_/*dt*) depend on (i) the rate of fertilizations, which is determined by the product of the gamete numbers of each type *x*_*t*_*y*_*t*_ (‘mass action’ [22,23]) multiplied by an encounter rate parameter *a* and (ii) on a per-gamete mortality rate *μ*_*x*_ or *μ*_*y*_, which is multiplied by the number of gametes of the corresponding type.

**Table 1.** Notation and definitions.

notation	name of parameter, variable or function	definition
*f*	fertilization function	function that mathematically describes the number of fertilizations resulting from a specific initial number of gametes (as well as other assumptions and parameters depending on the model)
*x*_0_=*x*, *y*_0_=*y*	initial gamete numbers	number of gametes of type *x* and type *y* (e.g. male and female gametes) that are released into the fertilization environment simultaneously, and that then either fuse with each other or die at rates specified in the model
*x*_*t*_, *y*_*t*_	gamete numbers remaining at time *t*	*x*_*t*_ and *y*_*t*_ denote the gamete numbers that remain at time *t* after gamete release, as they diminish over time due to fertilizations and gamete mortality. These are often solved first as an intermediate step towards the fertilization function *f*
*a*	gamete encounter rate parameter	parameter that determines the rate at which free pairs of gametes fuse with each other. This is analogous to e.g. the ‘aptitude for union of gametes’ [21] or ‘bimolecular reaction constant’ [10] in previous work
*μ*_*x*_, *μ*_*y*_	gamete mortality rate	per-gamete ‘mortality’ rate: all the ways in which gametes are removed from the pool of available gametes, except gamete unions. These can be time-dependent or -independent, and can also equal 0 (i.e. no gamete mortality). Depending on the requirements of the specific model, the mortality rates could also be defined to depend on gamete size
*T*	gamete lifespan	*T* denotes the fixed lifespan of gametes in model 1. This can also be interpreted as time-dependent, stepwise gamete mortality

The product of gamete numbers (*x*_*t*_*y*_*t*_) can also be interpreted as the remaining number of potentially colliding *pairs* of gametes at time *t* after gamete release. Note that *x*_*t*_ and *y*_*t*_ are functions of time *t*, and the gamete-specific mortalities can also be time-dependent if the gametes undergo some kind of senescence. The fertilization environment itself could be external or internal, and the gametes can originate from any number of potential parents, as long as they are synchronously released.

The aim is to use these equations to find explicit solutions for the total number of realized fertilizations. The details of how this is done depend on the specifics of the problem. Generally, some suitable intermediate result is solved from the initial value problem (equations (2.1)), which is then used to derive the actual fertilization function *f*. This general methodology will be used here to derive fertilization functions for specific, simple forms of gamete mortality that permit explicit solutions. Equations (2.1) can, in principle, be used with any form of gamete mortality, but closed-form solutions will not always be possible.

### 2.1 Model 1: time-dependent mortality, with fixed lifespan of gametes

First, I assume that standard deviation of individual gamete lifespans *T* is small, so that gamete mortality can be approximated with a step function (see [10]). Then *μ*_*x*_=*μ*_*y*_=0 for *t*≤*T*, and all gametes of type *x*, *y* or both lose their fertilization capability at time *T*. Equations (2.1) become

2.2 $$\left\{ \begin{array}{l} \frac{dx_{t}}{dt}=-ax_{t}y_{t}\\ \frac{dy_{t}}{dt}=-ax_{t}y_{t} \end{array}\right.\;\text{for}\;t\leq T\;\text{and}\;\left\{ \begin{array}{l} x_{0}=x\\ y_{0}=y \end{array}\right..$$

No more fertilizations take place after time *T*. Note that with this simple stepwise mortality it is not explicitly specified whether it is type *x* or type *y* gametes (or both) that die at time *T*. In a model, *T* could of course be explicitly defined as a function of, for example, the size of the smaller gamete type, which could reasonably be expected to have a shorter lifespan than the larger gametes.

To find the fertilization function *f*, first note that because gametes are only depleted due to fertilizations when *t*≤*T*, the Fisher condition [19] requires that *f*=*x*−*x*_*t*_=*y*−*y*_*t*_, and therefore *x*_*t*_=*y*_*t*_−*y*+*x*. Substituting this into equations (2.2), the number of remaining gametes at any time *t*≤*T* can be solved (using partial fractions [24]), which obtains

2.3 $$\left\{ \begin{array}{l} x_{t}=\frac{x(x-y)}{x-{\text{e}}^{at(y-x)}y}\\ y_{t}=\frac{y(y-x)}{y-{\text{e}}^{at(x-y)}x} \end{array}\right..$$

**Figure 1.**
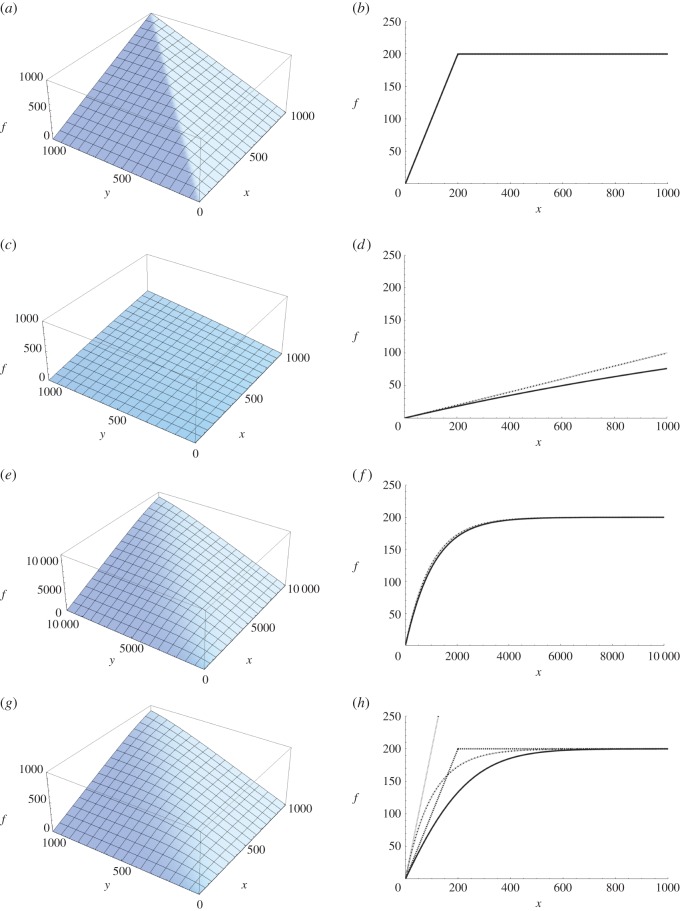
Various plots of equation (2.4). Plots of the other derived functions are visually quite similar and comparisons between the functions are much more convenient when three-dimensional rotation of the figures is possible. MATHEMATICA commands for plotting the figures are given in the electronic supplementary material. (*a*,*c*,*e*,*g*) The fertilization function *f* as a function of gamete numbers *x* and *y*. (*b*,*d*,*f*,*h*) Cross-sections of the three-dimensional plots, so that *y* is fixed at 200. The right-hand-side panels indicate that equation (2.4) is consistent with previously used fertilization functions under appropriate conditions (*b*,*d*,*f*), but is not always well approximated by any of them (*h*). In (*b*,*d*,*f*,*h*), the solid line corresponds to equation (2.4), while the dotted lines correspond to the relevant approximation (see §2.3, main text). Note that in panels (*b*) and (*f*) the solid and dashed lines are almost identical, and therefore difficult to tell apart. In (*a*), gamete limitation is low (*a*=*T*=1), and *f* is well approximated by min(*x*,*y*) (*b*). In (*c*), gamete limitation is quite strong (*a*=0.0005, *T*=1), and *aT xy* is a reasonable approximation of *f*(*d*). In (*e*), *a*=0.001 and *T*=1. Note that in (*f*), *x* is much larger than *y* over most of the range of *x*, and *f* is well approximated by the negative exponential function 200(1−e^−0.001*x*^). In (*g*), *a*=0.01 and *T*=1. *f* is poorly approximated by all of the approximations derived in section 2.3. In particular, (*h*) indicates that the approximations perform poorly when *x*≈*y*=200, i.e. when gamete dimorphism is low.

The actual fertilization function *f* is simply the difference between the initial number of gametes and that remaining unfertilized at time *T*:

2.4 $$f(x,y,a,T)=x-x_{T}=y-y_{T}=xy\frac{{\text{e}}^{aTx}-{\text{e}}^{aTy}}{x{\text{e}}^{aTx}-y{\text{e}}^{aTy}}.$$

(An alternative approach, leading to the same result, would be to calculate the integral $\int_{0}^{T}ax_{t}y_{t}dt$.) The symmetry of equation (2.4) already makes it clear that both gamete types are on an equal footing. The model also fulfills the Fisher condition [19], as the number of fertilizations for both sexes (*x* and *y*) is determined by the same equation, always yielding equal numbers of fertilized eggs and sperm. Plots of equation (2.4) with various parameter combinations are shown in figure 1.

If it is not necessary to independently model the collision rate *a* (which could in itself be a function of e.g. gamete size and speed [25,26]) and fertility time *T*, equation (2.4) can be simplified further. Noting that the encounter rate parameter *a* and the fertilization time parameter *T* only appear together as *aT*, they can be combined in a single parameter *φ*=*aT*, which controls the level of gamete limitation in the model; extremely low values of *φ* result in a very large fraction of gametes of both types remaining unfertilized, while very high values lead to essentially all gametes of the less numerous type being fertilized. Equation (2.4) could also be transformed into a function of the ratio of gamete numbers *r*, by defining *x*=*ry* or vice versa. The most useful form depends on the application and type of analysis.

### 2.2 Time-independent gamete mortality

In §2.1, gamete mortality was modelled in a stepwise fashion; fertilizations cease at a fixed time *T* after gamete release. In the opposite scenario, gamete mortality rate is constant from the moment of gamete release until there are no gametes left. It is useful to have analytical expressions for these two extremes, even though most intermediate forms of mortality cannot be solved in this way. I will next derive expressions for fertilization functions corresponding to three different cases of constant gamete mortality: (i) identical gamete mortality in the two sexes, (ii) gamete mortality in one sex only and (iii) independent, arbitrary gamete mortalities in the two sexes. Cases (i) and (ii) are essentially special cases of case (iii), but they permit much simpler solutions, and can therefore be useful for some purposes in themselves.

#### 2.2.1 Model 2: identical, time-independent mortality rates for the two gamete types

When mortality for male and female gametes is constant and identical, equation (2.1) with *μ*_*x*_=*μ*_*y*_=*μ* (where *μ* is time-independent) is used:

2.5 $$\left\{ \begin{array}{l} \frac{dx_{t}}{dt}=-ax_{t}y_{t}-\mu x_{t}\\ \frac{dy_{t}}{dt}=-ax_{t}y_{t}-\mu y_{t} \end{array}\right.\;\text{and}\;\left\{ \begin{array}{l} x_{0}=x\\ y_{0}=y \end{array}\right..$$

Subtracting the second equation from the first obtains a single differential equation for the *difference* between gamete numbers: d(*x*_*t*_−*y*_*t*_)/*dt*=−*μ*(*x*_*t*_−*y*_*t*_). This is a standard exponential decay equation, with the solution ($x_{t}-y_{t})=(x-y)e^{-\mu t}$. With this intermediate solution, it is possible to solve *x*_*t*_/*y*_*t*_, and then *x*_*t*_=(e^−*tμ*^*x*(*x*−*y*))/(*x*−e^−(*a*e^−*tμ*^(−1+e^*tμ*^)(*x*−*y*))/*μ*^*y*) and *y*_*t*_=(e^−*tμ*^*y*(*y*−*x*))/(*y*−e^−(*a*e^−*tμ*^(−1+e^*tμ*^)(*y*−*x*))/*μ*^*x*) from equation (2.5). Finally, the fertilization function is obtained as the total number of fertilizations from the integral $\int_{0}^{\infty }ax_{t}y_{t}dt,$ which yields (after simplification)

2.6 $$f(x,y,a,\mu )=\frac{\mu }{a}\ln\frac{x-y}{x{\text{e}}^{-(a/\mu )y}-y{\text{e}}^{-(a/\mu )x}}.$$

*a*/*μ* could again be replaced with a single parameter *φ*=*a*/*μ*, controlling the level of gamete limitation. Here, the average survival time of a single gamete is 1/*μ*, which is roughly analogous to the length of the fertilization period *T* in equation (2.4). However, the correspondence between *T* and 1/*μ* is not quite this simple. In the first model, fertilizations cease at a single moment *T*. The first model does not specify how this happens; it could be, in principle, because of all microgametes dying, all macrogametes dying, or both. Regardless of which option is true, the maximum time that any pair of gametes can be around for fertilizations is *T*. In the second model, each potential pair of gametes is composed of two gametes that both can die at any time, so the overall ‘pairwise mortality rate’ is 2*μ*, and average survival time of a potential gamete pair is 1/2*μ*. Therefore, a reasonably close correspondence could be expected between the two models if one used the same value of *a* in both, with *T*=1/2*μ* (but the results should not be expected to be identical even then, because of the difference in the mortality process).

A model where gametes of both types die continuously at exactly the same rate is of course somewhat limited. Therefore, I will next consider cases where gamete mortalities can differ between the sexes.

#### 2.2.2 Model 3: time-independent gamete mortality in one sex, no gamete mortality for the other sex

A relatively simple fertilization function can also be found for the case where gamete mortality is negligible for one sex, and constant for the other. This can be the case in, for example, internal fertilization, where retained gametes can be much better protected than transferred gametes, or even in external fertilization if one gamete type is much larger, and has negligible mortality compared with the other. Now equations (2.1) become

2.7 $$\left\{ \begin{array}{l} \frac{dx_{t}}{dt}=-ax_{t}y_{t}\\ \frac{dy_{t}}{dt}=-ax_{t}y_{t}-\mu y_{t} \end{array}\right.\;\text{and}\;\left\{ \begin{array}{l} x_{0}=x\\ y_{0}=y \end{array}\right..$$

Formally dividing the second equation by the first eliminates time from the differential equations: *dy*_*t*_/*dx*_*t*_=1+(*μ*/*a*)(1/*x*_*t*_). Rearranging and integrating yields *y*_*t*_−*y*=*x*_*t*_−*x*+(*μ*/*a*)ln(*x*_*t*_/*x*), which can be solved for *x*_*t*_: *x*_*t*_=(*μ*/*a*)W((*ax*/*μ*)e^(*a*(*x*−*y*+*y*_*t*_))/*μ*^). Here, *W* is the principal branch of the Lambert W-function [27]. Given that *x*-gametes only disappear due to fertilizations, while *y*-gametes die at a constant rate, the number of *x*-gametes remaining after all possible fertilizations have taken place is obtained simply by setting *y*_*t*_=0. By similar logic, the total number of fertilizations is the difference between the initial and final number of *x*-gametes

2.8 $$f(x,y,a,\mu )=x-\frac{\mu }{a}W\left(\frac{ax}{\mu }{\text{e}}^{a(x-y)/\mu }\right).$$

Again, *a*/*μ* could be replaced with a single gamete limitation parameter.

#### 2.2.3 Model 4: time-independent, arbitrary mortality rates for the two gamete types

The equations in §§2.2.1 and 2.2.2 are useful because of their relative simplicity. However, a more general fertilization function can be derived that allows for arbitrary (but still constant) mortality rates for the two gamete types that are independent of each other.

Now equation (2.1) is used as it is, with constant *μ*_*x*_ and *μ*_*y*_

2.9 $$\left\{ \begin{array}{l} \frac{dx_{t}}{dt}=-ax_{t}y_{t}-\mu_{x}x_{t}\\ \frac{dy_{t}}{dt}=-ax_{t}y_{t}-\mu_{y}y_{t} \end{array}\right.\;\text{and}\;\left\{ \begin{array}{l} x_{0}=x\\ y_{0}=y \end{array}\right..$$

To solve the equations, I begin again by eliminating time from the equations by dividing the first equation by the second: *dx*_*t*_/*dy*_*t*_=(*ax*_*t*_*y*_*t*_+*μ*_*x*_*x*_*t*_)/(*ax*_*t*_*y*_*t*_+*μ*_*y*_*y*_*t*_). As in §(2.2.2), *x*_*t*_ can then be solved as a function of *y*_*t*_. The fertilization function is then calculated as the integral $\int_{0}^{\infty }ax_{t}y_{t}dt$ using integration by substitution [24]; see the electronic supplementary material for details. The fertilization function is

2.10 $$f(x,y,a,\mu_{x},\mu_{y})=\int_{0}^{y}{\left(1+{\left[W\left(\frac{ax}{\mu_{y}}{\text{e}}^{(a(x-y+y_{t}))/\mu_{y}}{\left(\frac{y_{t}}{y}\right)}^{\mu_{x}/\mu_{y}}\right)\right]}^{-1}\right)}^{-1}dy_{t}.$$

Although it looks very different, equation (2.10) converges to equation (2.6) when *μ*_*x*_=*μ*_*y*_=*μ*, and to equation (2.8) when *μ*_*x*_=0 and *μ*_*y*_=*μ*. The price for the higher generality of equation (2.10) is that it looks relatively complicated, and involves an integral; I was unable to simplify this further in the general case. Nevertheless, it is a mathematical expression for the fertilization function, and its value can be easily calculated to high accuracy using mathematical software packages. It can also be differentiated (under the integral sign) like any other function, and therefore used as a component of optimization models [28]. Hence, equation (2.10) is not very different from elementary functions in terms of its applicability in modelling. The advantage of equation (2.10) is that the mortalities of the two gamete types can be defined to depend on their sizes or any other feature of the gametes, entirely independently of each other. This can be useful in any model where gamete size itself is either an evolving trait or a parameter in the model.

### 2.3 Consistency with previous models

It is illuminating to consider how these equations relate to earlier models of the fertilization process, by examining limits and linear approximations of the functions developed here.

Firstly, highly efficient fertilization conditions (i.e. no gamete limitation) can be examined by letting a→∞. It is relatively easy to calculate this limit for equations (2.4), (2.6) and (2.8). In all three cases, it can be shown that ${\text{lim}}_{a\to \infty }f=\text{min}(x,y)$. In other words, the function behaves as if gamete mortality had no effect and all gametes of the less numerous type (generally female gametes) get fertilized (figure 1*a*,*b*). This may seem obvious, but it is nevertheless a reassuring check of consistency in these models. I was unable to calculate this limit analytically for the more complicated equation (2.10), but numerical trials with large values of *a* indicate that the outcome is the same. The assumption that all female gametes are fertilized is in fact used in a vast number of evolutionary models, often implicitly (i.e. the fertilization function is explicitly or implicitly taken to be equal to the number of eggs). It also arises from negative exponential fertilization functions of the form *f*=*x*(1−e^−*cy*^) (e.g. [10,11]) when c→∞ (in these models, *x* corresponds to eggs, i.e. the less numerous gamete type).

A second, interesting, extreme case is that of very high gamete limitation: when the collision rate constant *a* is close to 0, large fractions of gametes of both types remain unfertilized. A convenient way to analyse this is to derive linear approximations (first order Taylor polynomials centred at *a*=0 (e.g. [23,24])) for each of the fertilization functions. This obtains *f*≈*aTxy*, *f*≈*a*/(2*μ*)*xy*, *f*≈(*a*/*μ*)*xy* and *f*≈(*a*/(*μ*_*x*_+*μ*_*y*_))*xy* for equations (2.4), (2.6), (2.8) and (2.10), respectively. These are all proportional to *xy*, i.e. the total number of fertilizations is approximately proportional to the product of the number of the two gamete types (figure 1*c*,*d*). Now the function behaves as if fertilizations were elastic collisions and gamete numbers were only depleted by gamete mortality. Fertilization functions of this form have in fact been used in models that either implicitly or explicitly assume strong gamete limitation (e.g. [13,29,30]). In line with these results, linearizing the negative exponential fertilization function *x*(1−*e*^−*cy*^) also results in *f*≈*cxy* for small values of *c*.

The fertilization functions derived here are also consistent with negative exponential fertilization functions. Equations (2.4) and (2.8) are derived under assumptions that are compatible with negligible egg mortality, a common assumption in earlier fertilization functions. Now, if we define *x*=*ry*, then *r*→0 corresponds to the typical assumption of very asymmetrical gamete numbers (*y*≫*x*). Setting *x*=*ry* and deriving first order Taylor polynomials for equations (2.4) and (2.8) centred at *r*=0 yields *f*≈*ry*(1−e^−*aTy*^)=*x*(1−e^−*aTy*^) and *f*≈*ry*(1−e^−(*a*/*μ*)*y*^)=*x*(1−e^−(*a*/*μ*)*y*^), respectively. Therefore, when sperm strongly outnumber eggs, and egg mortality is negligible, the models derived here are consistent with negative exponential fertilization functions (figure 1*e*,*f*). A similar result can be found for equation (2.10) when *μ*_*x*_=0, but not for equation (2.6) because the assumption *μ*_*x*_≪*μ*_*y*_ is broken.

## 3. Discussion

Many evolutionary models make use of some form of fertilization function, either explicitly or implicitly. The simplest fertilization function is one where all eggs are fertilized: in the notation of this article, *f*=min(*x*,*y*). This is equivalent to assuming that there is no sperm limitation (a→∞), and this assumption has been (quite reasonably) used in a vast number of influential models (e.g. many models of sex allocation [31], sperm competition [3] and the evolution of anisogamy [32]). A second relatively simple assumption is that the total number of fertilizations is proportional to the product of the number of sperm and the number of eggs: *f*∝*xy*. As shown above, this is equivalent to assuming that sperm limitation is very high, and a large proportion of both male and female gametes remain unfertilized. It has been used in models of anisogamy evolution that emphasize selection for high gamete encounter rates (e.g. [13,29,30]). However, it is debatable whether this limit is biologically realistic. While it is likely that essentially all eggs are fertilized in some species (a→∞), a scenario where the proportion of fertilized eggs approaches zero (*a*→0, as required for the approximations of very high gamete limitation) would usually not be able to sustain a population.

Nevertheless, fertilization processes under natural conditions can encompass a wide range of sperm limitation, particularly in external fertilizers [1,2,33]. Fertilization functions compatible with various levels of sperm limitation been derived (usually negative exponential functions, such as *f*=*x*(1−e^−*cy*^): [10,11]), and have been applied in evolutionary models of reproductive biology (e.g. [5–8]). These fertilization functions are suitable for situations where one gamete type strongly outnumbers the other, while mortality of the rarer type is negligible. In other words, they assume strong gamete dimorphism.

The value of the fertilization functions derived in this article is that they encompass the scenarios mentioned above (§2.3, but see below for some limitations), while extending the range of applicability to low dimorphism. Depletion of both gamete types due to fertilizations as well as gamete mortality is accounted for. These fertilization functions can be used as modular components in constructing evolutionary models where neither gamete type necessarily outnumbers the other by a large margin. This can be essential for evolutionary modelling of the origin as well as the consequences of gamete dimorphism, the defining feature of the two sexes. Such models should not pre-assume asymmetries between the sexes in the fertilization process, as it is the origin of these asymmetries that is being modelled.

More generally, the new fertilization functions make it possible to relatively easily combine two very important biological processes, sperm competition [3,4] and sperm limitation [1,2], in a single model without making assumptions about already diverged male and female gametes. To see how such a model could be constructed, consider a situation where *n*_*x*_ spawning females and *n*_*y*_ spawning males release their gametes simultaneously. An ESS approach [23,28] can then be used to analyse the simultaneous effect of gamete limitation and gamete competition as follows. Assume that resident females and males release *s*_*x*_ and *s*_*y*_ gametes per individual. Now, if a mutant female producing ${\hat{s}}_{x}$ gametes appears in the population, its fitness is affected by a gamete competition component ${\hat{g}}_{x}={\hat{s}}_{x}/({\hat{s}}_{x}+(n_{x}-1)s_{x})$ and a fertilization function component ${\hat{f}}_{x}=xy({\text{e}}^{aTx}-{\text{e}}^{aTy})/(x{\text{e}}^{aTx}-y{\text{e}}^{aTy})$, where $x={\hat{s}}_{x}+(n_{x}-1)s_{x}$; *y*=*n*_*y*_*s*_*y*_ and *a* and *T* are parameters that control the level of gamete limitation. Here I have used equation (2.4), but it could be replaced by any of the fertilization functions derived above. The product ${\hat{g}}_{x}{\hat{f}}_{x}$ then models the combined effect of gamete competition and gamete limitation on the mutant female's fitness, where the first component accounts for the relative share of fertilizations gained by the mutant female, and the second accounts for the total number of fertilizations. A similar function applies to males, obtained by simply switching all *x*s and *y*s. However, ‘male’ and ‘female’ are used here for convenience only; the equations are symmetrical, and any other label could be used to identify the two types. Note that conventionally, gamete competition is not considered to be important for females. This is accounted for in the model: it has been shown above that in the limit of no gamete limitation, *f*=min(*x*,*y*). Then, in the case of no gamete limitation, and assuming that *x* is the less abundant gamete type, the fertilization function and denominator of the gamete competition function for *x* cancel out, leaving only ${\hat{g}}_{x}{\hat{f}}_{x}={\hat{s}}_{x}/({\hat{s}}_{x}+(n_{x}-1)s_{x})\times ({\hat{s}}_{x}+(n_{x}-1)s_{x})={\hat{s}}_{x}$.

This approach forms a general model backbone for combining gamete competition and limitation, and it can be applied to various evolutionary problems where these processes might be important. For example, adding a third function that accounts for zygote survival, together with a gamete-size number trade-off would result in a relatively simple model of anisogamy evolution combining gamete competition and gamete limitation. It would be an interesting modelling problem for future work to reconstruct a previous, more complex model developed for the same purpose [18] using this simpler approach. The simpler, modular construction would also make it possible to explicitly compare the strength of the different components of selection [6]. Similarly, the fertilization functions developed here could be used to investigate the *consequences* of gamete dimorphism. The increasing discrepancy in gamete sizes and numbers has been argued to be a major driver in the differentiation of the sexes [34,35], and the fertilization functions derived here could potentially be used to model this evolutionary process, particularly in the early stages where gamete numbers of the two sexes may have been relatively similar.

The methods developed here may also be useful for other modelling purposes in evolutionary biology and ecology. Consider a mate search scenario, with resulting socially monogamous bonds (or, e.g. death after mating). Males and females are then analogous to male and female gametes. Free males and females are depleted by the process of forming pairs (analogous to fertilizations), or by mortality (which can be sex specific). Now the rate parameter *a* could be used to adjust the difficulty of finding mates under various population densities, simulating a type of Allee effect [36].

As a first step into developing generalized fertilization functions encompassing low gamete dimorphism, the models presented here contain some simplifying assumption. Previous models [10,11] have examined properties such as fertilization area of the egg, the collision rate constant (here, *a*) and the total probability of conception in more detail, whereas others have examined the effect of multiple sperm sticking to an (already fertilized) egg [10] and polyspermy [12]. These questions have not been examined here, as the focus of this article is on modelling the core fertilization process in a consistent way. However, some of these additional factors could be incorporated into the present model with ease; for example, the collision rate constant *a* could in itself be defined as a function of some properties of the gametes and the environment. Here, it is treated simply as a constant that describes the ‘aptitude for union of gametes’ [21]. Multiple sperm sticking to an egg, on the other hand, is a phenomenon that does not have a clear analogue when gamete dimorphism is very low, and sperm have not clearly diverged from eggs. This effect would have to be accounted for in a more complicated way, where the clumping together of multiple gametes depends on the level of gamete dimorphism. It should also be noted that, as in many other models, gamete numbers are modelled in a continuous (as opposed to discrete) fashion. This implies that using the functions for modelling scenarios where gamete numbers are extremely low should be approached with some caution.

## Supplementary Material

Mathematical details, and mathematica commands.

## Data accessibility

Details for the derivation of equation (2.10) and Mathematica code are included in the electronic supplementary material.
