# Supplementary material for: Models of fertilization kinetics
Source: R Soc Open Sci. 2015 Sep 16;2(9):150175. doi: 10.1098/rsos.150175 (PMC4593677; doi:10.1098/rsos.150175)
Supplement: Mathematical details, and mathematica commands. [file rsos150175supp1.pdf]

Supplementary material for ' Models of fertilization kinetics'

Jussi Lehtonen

1) Deriving equation (2.10)

Equation (2.9) yields $\frac{dx_t}{dy_t} = \frac{ax_t y_t + \mu_x x_t}{ax_t y_t + \mu_y y_t}$, which can be rearranged as

$\left(a + \frac{\mu_y}{x_t}\right) dx_t = \left(a + \frac{\mu_x}{y_t}\right) dy_t$. Integrating and solving for x_t, this obtains

$a(x - x_t) + \mu_y \ln \frac{x}{x_t} = a(y - y_t) + \mu_x \ln \frac{y}{y_t}$ and

$$x_t = \frac{\mu_y}{a} W\left(\frac{ax}{\mu_y} e^{\frac{a(x-y+y_t)}{\mu_y}} \left(\frac{y_t}{y}\right)^{\frac{\mu_x}{\mu_y}}\right).$$

The fertility function is then calculated as the integral $\int_0^\infty ax_t y_t dt$. Because x_t is known as a function of y_t, but not *t*, this is done using integration by substitution. Equation (2.9) yields $dt = \frac{dy_t}{-ax_t y_t - \mu_y y_t}$, and the new integration limits are $y_0 = y$ and $\lim_{t\to\infty} y_t = 0$ (assuming $\mu_y > 0$). Therefore, the integral transforms as follows:

$$\int_0^\infty ax_t y_t dt = \int_y^0 a \frac{\mu_y}{a} W\left(\frac{ax}{\mu_y} e^{\frac{a(x-y+y_t)}{\mu_y}} \left(\frac{y_t}{y}\right)^{\frac{\mu_x}{\mu_y}}\right) y_t \frac{dy_t}{-a\frac{\mu_y}{a}W\left(\frac{ax}{\mu_y}e^{\frac{a(x-y+y_t)}{\mu_y}}\left(\frac{y_t}{y}\right)^{\frac{\mu_x}{\mu_y}}\right)y_t - \mu_y y_t}$$

$$= \int_0^y \frac{W\left(\frac{ax}{\mu_y}e^{\frac{a(x-y+y_t)}{\mu_y}}\left(\frac{y_t}{y}\right)^{\frac{\mu_x}{\mu_y}}\right)}{1+W\left(\frac{ax}{\mu_y}e^{\frac{a(x-y+y_t)}{\mu_y}}\left(\frac{y_t}{y}\right)^{\frac{\mu_x}{\mu_y}}\right)} dy_t$$

$$= \int_0^y \left(1 + \left[W\left(\frac{ax}{\mu_y} e^{\frac{a(x-y+y_t)}{\mu_y}} \left(\frac{y_t}{y}\right)^{\frac{\mu_x}{\mu_y}}\right)\right]^{-1}\right)^{-1} dy_t$$

which is equation (2.10).

2) Mathematica commands

These commands are compatible with Mathematica 9.0; compatibility with other versions is not guaranteed.

Mathematica commands for defining the fertility functions derived in the main text:

Equation (2.4)
```
f1[x_,y_,a_,t_]=x y (Exp[a t x]-Exp[a t y])/(x Exp[a t x]-y Exp[a t y])
```

Equation (2.6)
```
f2[x_,y_,a_,u_]=u/a Log[(x-y)/(x Exp[-a y/u]-y Exp[-a x/u])]
```

Equation (2.8)
```
f3[x_,y_,a_,u_]=x-u/a ProductLog[a x/u Exp[a (x-y)/u]]
```

Equation (2.10)
```
f4[x_,y_,a_,u1_,u2_]:=Integrate[(1+(ProductLog[a x/u2 Exp[a (x-y+z)/u2] (z/y)^(u1/u2)])^(-1))^(-1),{z,0,y}]
```

Mathematica commands for all the panels of figure 1. Replace ‘f1’ with the appropriate function to plot figures for the other versions of the fertility function. Note the differences in parameterization (see main text), and that f4 requires one more parameter than the other functions. Font settings may need to be adjusted depending on the display size and settings.

a)
```
Plot3D[f1[x,y,1,1],{x,0,1000},{y,0,1000},BaseStyle->{FontWeight->"Bold",FontSize->50},ViewPoint->{-3,-2,2.3},ImageSize->Full,PlotRange->{{0,1000},{0,1000},{0,1000}}]
```

b)
```
Plot[{f1[x,200,1,1],Min[x,200]},{x,0,1000},PlotRange->{{0,1000},{0,250}},ImageSize->Full,BaseStyle->{FontWeight->"Bold",FontSize->50},PlotStyle->{Directive[Black,Thickness[0.005]],Directive[Black,DotDashed,Thickness[0.005]],Directive[Black,DotDashed,Thick]}]
```

c)
```
Plot3D[f1[x,y,0.0005,1],{x,0,1000},{y,0,1000},BaseStyle->{FontWeight->"Bold",FontSize->50},ViewPoint->{-3,-2,2.3},ImageSize->Full,PlotRange->{{0,1000},{0,1000},{0,1000}}]
```

d)
```
Plot[{f1[x,200,0.0005,1],0.0005 200 x},{x,0,1000},PlotRange->{{0,1000},{0,250}},ImageSize->Full,BaseStyle->{FontWeight->"Bold",FontSize->50},PlotStyle->{Directive[Black,Thickness[0.005]],Directive[Black,DotDashed,Thickness[0.005]],Directive[Black,DotDashed,Thick]}]
```

e)
```
Plot3D[f1[x,y,0.001,1],{x,0,10000},{y,0,10000},BaseStyle->{FontWeight->"Bold",FontSize->50},ViewPoint->{-3,-2,2.3},ImageSize->Full,PlotRange->{{0,10000},{0,10000},{0,10000}}]
```

f)
```
Plot[{f1[x,200,0.001,1],200 (1-Exp[-0.001 x])},{x,0,10000},PlotRange->{{0,10000},{0,250}},ImageSize->Full,BaseStyle->{FontWeight->"Bold",FontSize->50},PlotStyle->{Directive[Black,Thickness[0.005]],Directive[Black,DotDashed,Thickness[0.005]],Directive[Black,DotDashed,Thick]},LabelStyle->(FontFamily->"Times New Roman")]
```

g)
```
Plot3D[f1[x,y,0.01,1],{x,0,1000},{y,0,1000},BaseStyle->{FontWeight->"Bold",FontSize->50},ViewPoint->{-3,-2,2.3},ImageSize->Full,PlotRange->{{0,1000},{0,1000},{0,1000}}]
```

h)
```
Plot[{f1[x,200,0.01,1],200 (1-Exp[-0.01 x]),0.01 200 x,Min[x,200]},{x,0,1000},PlotRange->{{0,1000},{0,250}},ImageSize->Full,BaseStyle->{FontWeight->"Bold",FontSize->50},PlotStyle->{Directive[Black,Thickness[0.005]],Directive[Black,DotDashed,Thickness[0.005]],Directive[Black,Dotted,Thickness[0.005]],Directive[Black,Dashed,Thickness[0.005]]},LabelStyle->(FontFamily->"Times New Roman")]
```
